# Stretched to the Max: The Successful Medical Management of Ogilvie Syndrome in a Pediatric Patient

**DOI:** 10.7759/cureus.14506

**Published:** 2021-04-15

**Authors:** Stephany Chiacchio, Merlin C Lowe

**Affiliations:** 1 Pediatrics/Emergency Medicine, The University of Arizona, Banner-Diamond Children's Medical Center, Tucson, USA; 2 Pediatrics/Hospital Medicine, The University of Arizona, Banner-Diamond Children's Medical Center, Tucson, USA

**Keywords:** ogilvie's syndrome, acute colonic pseudo-obstruction, inpatient pediatrics, erythromycin

## Abstract

Ogilvie syndrome, or acute colonic pseudo-obstruction, is a rare disease in adults, and it is seldom seen in pediatric patients. It was first described in 1948 by Dr. William Ogilvie. Unless promptly recognized and treated, it carries the risk of colonic ischemia and perforation. In this report, we present the case of a 10-year-old patient who developed Ogilvie syndrome and was successfully treated with conservative medical management including bowel rest, rectal decompression, along with the addition of erythromycin. The patient responded well to the treatment and was able to be discharged home without event.

## Introduction

Ogilvie syndrome, also known as acute colonic pseudo-obstruction, was first described by Sir William Ogilvie in 1948 [1]. His case report involved two patients with retroperitoneal tumors that were not causing any mechanical obstruction. Most cases of Ogilvie syndrome in the medical literature involve adults who are either critically ill or in post-surgical situations, who present with signs and symptoms of bowel obstruction and are found to have a localized colonic ileus upon investigation. Pediatric presentation is extremely rare, but it important to recognize it promptly due to the rare but serious potential for colonic perforation. In this report, we discuss the case of a 10-year-old boy who developed Ogilvie syndrome after admission for a bowel cleanout to treat acute-on-chronic fecal impaction.

## Case presentation

**Figure 1:**
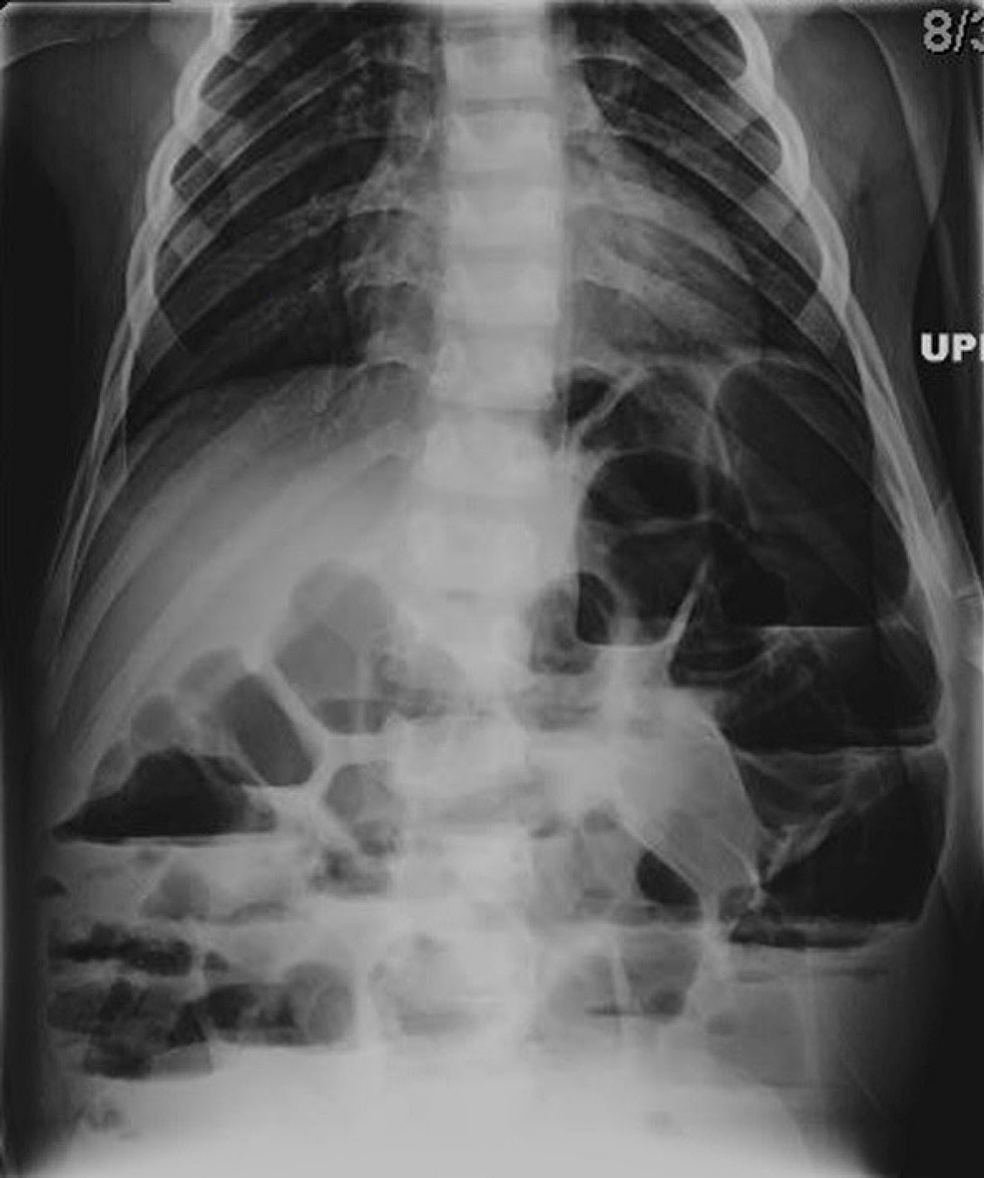
Upright abdominal X-ray showing dilated colon

A 10-year-old boy with a history of chronic constipation presented to the emergency department with fecal impaction. He was admitted and started on oral polyethylene glycol 3350 (PEG) for bowel cleanout. After multiple doses and successful bowel movements, the patient developed significant abdominal distention, nausea, and vomiting. Abdominal roentgenograph (AXR) showed dilated large bowel and air-fluid levels (Figure 1).

**Figure 2:**
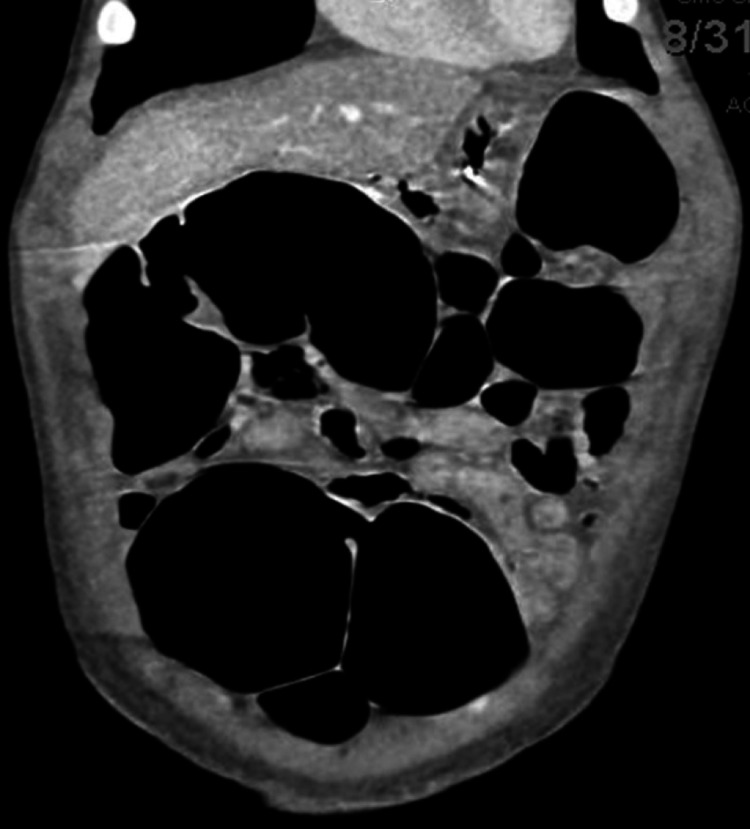
Abdominal sagittal CT scan image showing colonic distention CT: computed tomography

A nasogastric (NG) tube was placed; the patient was made nil per os (NPO) and laxatives were discontinued. A CT again showed dilated large bowel, without evidence of mechanical obstruction (Figure 2).

His distention slowly improved spontaneously, and he was started on clear liquids by mouth. However, despite the improvement, he again developed significant abdominal distention and was no longer passing stool. A repeat AXR revealed localized large bowel distention. At this point, the patient was made NPO again; a rectal tube was placed for decompression, erythromycin was started, and a barium enema was performed to rule out a very distal mechanical obstruction. The barium enema confirmed no mechanical obstruction. The pediatric gastroenterologist (GI) was consulted, who endorsed the current conservative management. Repeat films showed improvement in large bowel distention. The rectal tube was removed, and the patient's diet was normalized slowly. The patient was discharged on a high-fiber diet, PEG, erythromycin, and advice for a pediatric GI follow-up.

## Discussion

Ogilvie syndrome is quite rare in pediatric patients. The exact incidence of the condition is unknown, as most cases go unrecognized. It is thought to affect males more than females and usually occurs in the late middle age [2]. The causes of Ogilvie syndrome are not well established. Dysregulation of the autonomic nervous system is currently thought to be the most likely cause. Sir William Ogilvie first described it as sympathetic deprivation, meaning parasympathetic nerves (PNS) were acting unopposed [1]. He postulated that the tumors seen in his patients invaded the celiac plexus leading to sympathetic deprivation. However, with our current knowledge of the autonomic nervous system, we know that the parasympathetic system increases gut motility while the sympathetic nervous system decreases it, indicating that the cause would be a down-regulation of PNS and/or sympathetic overdrive [3].

Our patient’s history was consistent with chronic constipation leading to acute impaction, and in children, such diseases as hypothyroidism and Hirschsprung’s should be excluded. These had been ruled out for our patient in prior evaluations. We may hypothesize that his chronic constipation may have been secondary to autonomic dysfunction, which was likely multifactorial in this case, ultimately leading to his presentation with an acute colonic pseudo-obstruction.

Complications associated with Ogilvie syndrome can include perforation or ischemia. Perforation is rare and occurs in 1-3% of patients with Ogilvie [2]. Several studies suggest the risk of perforation appears to increase when the colonic diameter is greater than 9 cm, although some may argue that up to 12 cm of perforation still represents a low risk [4]. One study also concluded that the duration of colonic distention was correlated with a risk of perforation and not the actual size of the colon [5]. These findings were generated from adult studies and may not be valid for pediatric patients. Due to the condition's rarity, studies on Ogilvie syndrome in pediatric patients are scarce in the literature.

The treatment of the condition can be supportive, pharmacological, or may require surgical decompression. IV fluid resuscitation and electrolyte correction should be initiated in patients. A rectal tube can be placed to allow for decompression, which can minimize the risk of vascular compromise and colonic perforation. In our case, our initial efforts included placing an NG tube, which, while helping with abdominal distention, ultimately failed to decompress the distal colon. Our research on pharmacological agents has revealed that neostigmine may have the best evidence as an efficacious medication [6]. Neostigmine is an anticholinesterase (Ach) inhibitor, which allows for Ach to be available at the synaptic level. Its side effects include bradycardia, salivation, and diaphoresis. Since these trials were performed in adults, we opted not to try neostigmine as the first-line treatment option. Instead, we utilized another pro-kinetic agent, erythromycin, which had been described as a treatment option for Ogilvie syndrome in a pediatric patient [7]. With colonic decompression via rectal tube, erythromycin intake, and bowel rest, our patient's condition slowly improved and he eventually had a resolution of all his symptoms. Unfortunately, due to the limited availability of data on Ogilvie syndrome in pediatric patients, the risk of recurrence of the same is unknown.

## Conclusions

Our patient represents one of only a few cases of Ogilvie syndrome reported in a pediatric patient. Ogilvie syndrome is quite rare in pediatric patients and its exact incidence is unknown. Our management of the condition included fluid resuscitation, bowel rest, rectal tube for decompression, and erythromycin. The outcome was favorable, and the patient was ultimately discharged home on a bowel regimen and with advice to continue with erythromycin, providing further evidence that conservative management may be appropriate before surgical intervention is utilized. Pediatric hospitalists and pediatric emergency medicine physicians should be aware of Ogilvie syndrome in order to implement treatment rapidly. Rectal decompression and, possibly, prokinetic agents such as erythromycin can help prevent the rare but serious complication of colonic perforation or ischemia.

## References

[1] Ogilvie H (1948). Large-intestine colic due to sympathetic deprivation; a new clinical syndrome. Br Med J.

[2] (2021). Ogilvie syndrome. https://rarediseases.org/rare-diseases/ogilvie-syndrome/.

[3] Maloney N, Vargas HD (2005). Acute intestinal pseudo-obstruction (Ogilvie's syndrome). Clin Colon Rectal Surg.

[4] Vanek VW, Al-Salti M (1986). Acute pseudo-obstruction of the colon (Ogilvie's syndrome). An analysis of 400 cases. Dis Colon Rectum.

[5] Johnson CD, Rice RP, Kelvin FM, Foster WL, Williford ME (1985). The radiologic evaluation of gross cecal distension: emphasis on cecal ileus. AJR Am J Roentgenol.

[6] Ponec RJ, Saunders MD, Kimmey MB (1999). Neostigmine for the treatment of acute colonic pseudo-obstruction. N Engl J Med.

[7] Jiang DP, Li ZZ, Guan SY, Zhang YB (2007). Treatment of pediatric Ogilvie's syndrome with low-dose erythromycin: a case report. World J Gastroenterol.

